# Early Predictors of Renal Dysfunction in Egyptian Patients with β-Thalassemia Major and Intermedia

**DOI:** 10.4084/MJHID.2014.057

**Published:** 2014-09-01

**Authors:** Azza A.G. Tantawy, Nagham El Bablawy, Amira A.M. Adly, Fatma S.E. Ebeid

**Affiliations:** 1 Departments of Pediatrics, Ain Shams University, Cairo, Egypt

## Abstract

**Background:**

Better survival of thalassemia patients allowed previously unrecognized renal complications to emerge.

**Objectives:**

Assess prevalence and early predictors of renal dysfunction in young β-thalassemia major (β-TM) and intermedia (β-TI) patients.

**Subjects:**

66 β-TM (group I), 26 β-TI (group II) Egyptian patients and 40 healthy controls.

**Methods:**

Clinical assessment and laboratory data including kidney and liver function tests, such as serum ferritin, serum bicarbonate, plasma osmolality and urinary total proteins, microalbuminuria (MAU), N-acetyl-β-D-glucosaminidase (NAG), retinol binding protein (RBP), α-1 microglobulin, bicarbonate, osmolality, creatinine clearance (CrCl), % fractional excretion of bicarbonate (% FE-HCO3).

**Results:**

The prevalent renal abnormality was proteinuria (71%), followed by increased urinary level of RBP (69.4%), NAG (58.1%), α-1 microglobulin (54.8%) and microalbuminuria (29%) and also decreased urinary osmolality (58.1%). CrCl was a better assessment of renal function and significantly lowered in thalassemia patients. Tubular dysfunctions were more significant in splenectomized β-TM patients who showed more elevation of NAG and α-1 microglobulin and lower urinary osmolality. NAG, RBP and α-1 microglobulin were negatively correlated with CrCl and positively correlated with serum ferritin and urinary total protein. Z-score analysis for identifying patients with renal dysfunction proved superiority of urine total protein and RBP. Comparative statistics of different frequencies revealed significant difference between the urinary total protein and both MAU and % FE-HCO3.

**Conclusion:**

Asymptomatic renal dysfunctions are prevalent in young β-TM and β-TI patients that necessitate regular screening. Urinary total protein and RBP may be cost-effective for early detection.

## Introduction

Improvement of survival in patients with β-thalassemia has allowed several clinical morbidities to manifest, including renal complications.^1^ Renal dysfunction in these patients is not fully understood and seems to be multifactorial; attributed mainly to long-standing anemia, chronic hypoxia, iron overload and toxicity of iron chelators.^2^

Evidence of proximal tubular damage is observed in patients with β-TM. Low-molecular-weight proteinuria is found in almost all patients. Moreover, several studies report increased urinary excretion of several markers of proximal tubular damage in a considerable number of patients with β-TM. including N-acetyl-b-D-glucosaminidase (NAG) and b2-microglobulin (up to 60%); calcium (approximately 13%), phosphate and magnesium (about 9%), uric acid (30%–40%), amino acids (approximately 30%), and malondialdehyde derived from the destruction of membrane lipids by peroxidation.^3^

Assessment of tubular function involves evaluation of functions of the both proximal tubule (tubular handling of sodium, glucose, phosphate, calcium, bicarbonate and aminoacids) and distal tubule (urinary acidification and concentration).^4^ The integrity of renal tubules can be examined through urinary measurement of one or more proteins of low molecular weight (LMW), as α-1 microglobulin (31 KD) and retinol binding protein (RBP) (22KD).^5^ Both these parameters are freely filtered through the glomeruli and reabsorbed by proximal convoluted tubules.^6^ Although the enzyme NAG is of high molecular weight (140 KD), it is considered as a marker of renal tubular function mainly because it is secreted by tubular epithelium and its measurement has been undertaken in a variety of diseases associated with renal injury.^7^

Glomerular integrity can be assessed by measuring the concentration of urinary protein that is predominantly retained by the healthy glomerulus. The proteinuria of glomerular origin is an independent risk factor that strongly predicted those patients at great risk of progressive loss of renal function.^8^

Early identification of patients, at high risk of developing renal failure, is of great importance as it may allow specific measures to delay the progression of renal damage and thus reduce the incidence of end-stage renal failure and mortality.^9^ We aimed to evaluate the renal function status in β-TM and β-TI patients through comprehensive laboratory testing to entail the proper site of the lesion, either glomerular or tubular and assess prevalence and severity of renal glomerular and tubular dysfunction, and determine its early predictors.

## Subjects

A cross-sectional, case-control study has been performed including 66 patients with β-TM (group I) and 26 β-TI (group II) attending the Pediatric Hematology Clinic, Ain Shams University. Their diagnoses were based on hematological parameters and hemoglobin electrophoresis and were classified into:

[one] Group-I comprised 66 patients with β-TM, aged 2.5 – 13 years (mean 6.8 ±3.3 years), 42 males and 24 females, they were subdivided into two subgroups; Group-Ia (splenectomized group) (n°40) Group-Ib (non-splenectomized group) (n° 26). They received approximately 15 ml of packed red blood cells per kilogram body weight at each transfusion every 2–3 weeks interval, to maintain their hemoglobin levels around 8 g/dl.
[two] Group-II comprised 26 patients β-TI, aged 2.5 – 16 years (mean 7.6±4.7 years), 18 males and eight females. They were intermittently transfused, and their transfusion therapy was initiated mainly for failure to thrive in childhood, persistent worsening of their anemia, or development of complications during the course of the disease.

The study also included 40 healthy children, age and sex matched, as control group (Group-III).

The study was approved by The Medical Ethics Committee of Human Experimentation of Ain Shams University. Informed consent was obtained from parents or legal guardians.

## Methods

All recruited children were subjected to a detailed history with emphasis on disease duration, transfusion and chelation history, splenectomy status and symptoms suggesting renal abnormalities. A clinical examination stressing on anthropometric measures, and including echography for abdominal and renal assessment, was conducted on patients. Furthermore, they were subjected to comprehensive laboratory investigations including:

1. Hematological assessment included complete blood count (CBC), hemoglobin analysis with HPLC (high-performance cation exchange liquid chromatography), and indirect bilirubin dosage, as a marker of hemolysis.
2. Serum creatinine and blood urea nitrogen (BUN) were classified according to standard normal ranges for age and sex.^10^ Creatinine clearance (CrCl) was calculated from 24-h urine specimens using the standard formula: (U) * (V/P)/(1.73/BSA), where U = 24-h urine creatinine concentration, V = (total volume of urine collected)/(hours of urine collection * 60 min), P = serum creatinine and BSA = body surface area (m2).
3. Total protein, alanine aminotransferase (ALT), aspartate aminotransferase (AST), Hepatitis markers including hepatitis B surface antigen (HBsAg) and hepatitis C virus (HCV) antibody were measured Serum ferritin was measured using immulite instrument, based on two-site chemiluminescent-immunometric assay.^11^
4. Serum and urine bicarbonate were measured by Synchron CX7 auto-analyzer, applying a potentiometric principle.^12^ % FE-HCO3 was calculated using the standard formula: (urinary bicarbonate/serum bicarbonate) *100/(urinary creatinine/serum creatinine)
5. Serum and urine osmolality were measured by Osmotat030, based on lowering sample temperature below its freezing point-7°C. Urine/serum osmolality ratio was then calculated.
6. Colorimetric estimation of total urinary protein was done using “pyrogallol red” (DiaSys, Diagnostic Systems International, USA), where proteins-dye form a red complex measured at 600nm.^13^
7. Microalbuminuria was measured by SERA-PAK immuno-microalbumin kit (Bayer Corporation, Benedict, Eve, Tarry Town, NY, USA). The samples were mixed with specific antibody, which had polyethylene glycol as an enhancer and then incubated. Precipitates form a turbidity, which is directly related to the albumin concentrations, and measured at 340 nm.^14^
8. N-acetyl-BD-glucosaminidase (NAG) was measured by a colorimetric assay kit. NAG hydrolyzes the substrate 3-cresolsulfonphthaleinyl-N-acetyl-BD-glucosaminidase-sodium salt with the release of 3-cresolsulfonphtalein sodium salt (3-cresol purple) which is finally measured photo-metrically at 580nm.^15^
9. Retinol binding protein (RBP) was estimated by applying enzyme-linked immune-sorbent assay (ELISA) method. The diluted urine samples were firstly incubated into microplate wells pre-coated with an antibody specific for RBP. Then the horseradish peroxidase conjugated antibody was added and further incubated. Following a final washing step, substrate solution was incubated into the wells resulting in a colored product and hydrochloric acid, a stopping solution, was added. The color was measured at 450 nm, and its intensity is proportional to the amount of RBP present in the sample.^16^
10. Alpha-1 microglobulin assay was estimated by applying an indirect solid phase enzyme immunoassay kit was used. Calibrators, Controls and pre-diluted urine samples were firstly incubated into microplate wells pre-coated with highly purified anti-α1 microglobulin. Then the horseradish peroxidase conjugate antibody was pipetted into the wells to form the sandwich complexes. A chromogenic substrate solution was dispensed and incubated and then hydrochloric acid, a stopping solution, was added. The optical density was read at 450 nm, and dichromatic measurement with a 600–690 nm reference reading was recommended.^17^

## Statistical Methods

Statistical analysis was done on a personal computer with SPSS, version 9.05, 1998, USA. The mean, standard deviation and range were calculated. Student t test was performed, for comparative analysis, between groups and Pearson’s correlation coefficient (r), was applied for the correlation study. Frequency of renal abnormalities among patients was calculated at cut-off levels corresponding to mean ± 2SD of healthy controls, and X^2^ test was applied to compare different frequencies. Moreover, Z-score analysis was performed to find out which markers were powerful in identifying patients with renal impairment. Z-score describes the number of SDs, the parameter in a specified group (renal affected patients) away from the negative group (normal renal function patients).

## Results

The demographic characteristics and the biochemical parameters of the patients enrolled were presented in Table 1. The thalassemic patients demonstrated high level of hepatitis infection, fifteen patients (16%) had hepatitis infection; eleven of them were anti-HCV positive, and four were HBsAg positive. Also, the thalassemic patients showed a significant elevation of the liver transaminases (ALT, AST), and this was more prominent in splenectomized β-TM who also showed a significant lower serum albumin and total protein levels. Fifty percent of β-TI patients were on no iron chelation therapy compared to only 5% in β-TM patients (p<0.0001). Deferiprone was the mostly used iron chelator. It was used as a single chelator by 35% of β-TM patients and 25% of β-TI patients. 20% of β-TM patients and 15% of β-TI patients were deferoxamine only. 40% of β-TM patients were on combined deferiprone and deferoxamine chelation therapy compared to only 10% of β-TI patients. None of our patients was on the iron chelator deferasirox.

Although serum creatinine and BUN were not statistically different between thalassemic patients and controls (Table 1), corrected creatinine clearance were significantly lowered in both groups I and II (P<0.05 and P <0.01, respectively) (Table 2). Urinary total protein and microalbuminuria were significantly increased in all thalassemic (β-TM and β-TI) patients (P<0.01).

Urinary tubular markers (NAG, RBP and α-1 microglobulin), were significantly higher in all thalassemic (β-TM and β-TI) patients compared to controls. Moreover, β-TM patients showed significantly higher value in compare to β-TI patients (P<0.01, P<0.05 respectively). Calculated % Fe- HCO3, urine osmolality, and U/S osmolality were significantly different in all thalassemic patients versus controls and the effect was more prominent in β-TM patients (P<0.01) than in β-TI patients (P< 0.05) (Table 2).

Correlation study showed that markers of proximal tubular function (NAG, RBP and α-1 microglobulin) were negatively correlated with CrCl (P<0.01, P<0.05, P<0.01 respectively), and were positively correlated with serum ferritin (P<0.01) and urine total protein (P<0.01, P<0.05, P<0.01 respectively) (Table 3).

The frequencies of renal abnormalities were calculated in the studied patients at cut-off levels corresponding to mean±2SD of healthy controls. The most-prevalent renal abnormality was the proteinuria (71%), followed by increased urinary level of RBP (69.4%), NAG (58.1%), α-1 microglobulin (54.8%) and microalbuminuria (29%) and also decreased urinary osmolality (58.1%) (Table 4).

Comparative statistics of the calculated frequencies (Chi-Square test) revealed that there was a significant difference between the urinary total protein and both MAU (X^2^= 22.7; P<0.01) and % FE- HCO3 (X^2^= 18.6; P<0.01) (Table 5)

The Z-score analysis for identifying of patients with renal dysfunction proved superiority of both urine total protein and RBP as powerful markers compared to the other studied parameters (Table 6).

## Discussion

Although advances in the care of patients with β-thalassemia translate into better survival, this success allowed previously unrecognized complications to emerge that included several renal abnormalities.^18^ β-thalassemia major, the severe form, present in the first year of life with profound anemia and subsequently require regular blood transfusions for survival, as well as iron chelation therapy to treat iron overload and prevent end-organ damage.^19^

β-thalassemia intermedia present later in life with a milder form of anemia and remain largely transfusion-independent phenotype.^20^ They develop considerable iron overload due to increased intestinal iron absorption triggered by the ongoing ineffective erythropoiesis.^21^ We demonstrated elevated levels of serum ferritin in thalassemic patients reflecting high iron deposition in both β-TM and β-TI, but it was significantly higher in splenectomized β-TM. Serum ferritin was positively correlated with the studied markers of tubular function, and this may provide evidence for the suggested theory of participation of free iron in proximal tubular dysfunction although the exact mechanism was not investigated in this work.

Renal tubular dysfunctions have been described previously with increasing frequency in patients with β-TM.^22^ Many studies have demonstrated a proximal tubular damage, leading to increased urinary excretion of NAG, beta-2 microglobulin, and LMW proteins.^2^,^23^ The contemporary presence of proteinuria, aminoaciduria, low urine osmolality^24^ and also hyperuricosuria (54%) with renal uric acid wasting^25^ suggest a more complex damage. Our study demonstrates a high frequency of renal abnormalities in the studied children with β-TM and β-TI. The most frequent renal abnormality was proteinuria (71%), followed by increased urinary level of RBP (69.4%) and NAG (58.1%), decreased urinary osmolality (58.1%), presence of α-1 microglobulin (54.8%) and microalbumin (29%); these data suggest complex renal alterations in thalassemic patients, even if in some patients the tubular dysfunction could be prevalent.

The underlying mechanism for renal dysfunctions in thalassemia patients is not clear. They seem to be multifactorial, attributed mainly to include longstanding anemia, chronic hypoxia, iron overload;^24^ the presence of excess unpaired globin chains and high non-hemoglobin iron content, represent a potential transitional pool of free iron that may play a major role in lipid peroxidation.^26^,^27^

Chelation therapy may also affect renal function in thalassaemia patients. Deferoxamine does not affect the kidneys unless it is given intravenously, especially at high doses.^28^ The new oral iron chelator, deferasirox, can cause increases in serum creatinine, proteinuria, and even renal failure.^29^ Awareness of underlying renal dysfunction in thalassaemia can inform decisions now about the use and monitoring of iron chelation.^30^ Most of our β-TM patients were well chelated, forty percent of them were on combination therapy deferoxamine and deferiprone and none of the studied patients was treated with deferasirox, due to its high cost.

In considering the potential mechanisms of renal injury, anemia and associated potential chronic hypoxia could lead to activation of the oxidative stress cascade,^31^ and may also lead to changes in the morphology of cells in terms of size and vascular supply.^32^ A good correlation between the severity of anemia and markers of tubular abnormalities are reported in patients with β-TM.^33^ Our patients were transfused at low hemoglobin level with mean hemoglobin around eight (g/dl). The scarcity of blood available for the patients justify this approach and may reflect the negative cultural attitude towards blood donation and limited resources of public health system of developing country like Egypt. This level of anaemia and consequent hypoxia may explain the high frequency of renal abnormalities in the studied children with β-TM and β-TI.

According the results of this study, abnormalities in GFR are evident in patients with thalassemia, as demonstrated by occurrence of an hyperfiltration.^2^ Anemia may reduce systemic vascular resistance, by determining a hyperdynamic circulation, that increases renal plasma flow and GFR.^34^ That eventually can lead to stretching of the glomerular capillary wall and subsequent endothelial and epithelial injury, which induce transudation of macromolecules into the mesangium and consequent glomerular dysfunction.^35^ In the long-term, such changes may lead to a progressive decline in GFR.^36^ In the present work creatinine clearance was the best assessment of renal total function and was significantly lowered in thalassemic patients.

The defect of concentrating ability could be caused by increased blood flow through the vasa recta that could disturb the countercurrent multiplication effectiveness.^37^

The results of the present work demonstrated a maximal lowering of urine osmolality in β-TM patients who had more degree of anemia that is known to have a hyperperfusion effect. Moreover, the significant negative correlation of serum ferritin with urine osmolality would support the previous hypothesis of iron deposition in renal tubules.

Fractional excretion of bicarbonate is a marker of proximal tubular handling of bicarbonate.^4^ Preliminary evaluation of bicarbonate generation of the kidneys revealed a significant elevation of % FE-HCO3 in the thalassemic patients (32.2%) compared to healthy controls. That suggests a distal tubular defect, whereas a major portion of patients had elevated LMW proteins as RBP (69.4%) and hyposthenuria (54.8%). That suggests that distal tubular dysfunction is a late sequela of the renal tubular involvement in thalassemic patients who can show an intact handling of bicarbonate

Examining the pattern of tubular dysfunction among our β-TM patients revealed that the degree of defect is more marked in splenectomized patients than in non-splenectomized group. Indeed, prominent elevation of NAG and α-1 microglobulin, lowering of urine osmolality and urine/serum osmolality and also pronounced elevation of serum ferritin were found more frequently in splenectomized patients. Ongazyooth and his colleagues also established that tubular defects were more prominent in splenectomized patients who had higher levels of serum.^37^

The splenectomized β-TM patients showed evidences of liver impairment as manifested by elevated total bilirubin, ALT and lowered total proteins and albumin. The contribution of viral hepatitis infection to liver impairment cannot be excluded. Hepatitis B and C, should be considered also as potential causes of renal disease,^25^ especially when the thalassemic patients demonstrated high level of hepatitis infection. Fifteen patients (16%) of the present series had hepatitis infection; eleven of them were anti-HCV antibody positive and four were HBsAg positive. However, all the positive patients had normal baseline renal function, and none of them had elevated serum creatinine above upper normal limit or had a history of nephrotic syndrome, hypertension or diabetes.

Several researches have demonstrated improved sensitivity and specificity of measurement of urinary albumin as this is the predominant urinary protein, for evaluation of glomerular permeability.^38^,^39^ Our study proved the superiority of both urinary total protein and urinary RBP as a powerful marker for identifying patients with renal dysfunction and so highlighted the importance of laboratory assay, in the future screening and follow-up programs.

## Conclusions

Asymptomatic renal dysfunctions both glomerular and tubular are prevalent in young β-TM and β-TI patients that then necessitate a regular screening and follow-up. Urinary total protein and urinary RBP may be cost-effective markers for early detection of renal dysfunction.

## Study Limitations

We did not study uricemia and uricosuria in our thalassemia patients. Further longitudinal prospective studies on a larger number of patients is needed to prove the predictive value of the studied markers.

## Figures and Tables

**Table 1:** The demographic characteristics and biochemical parameters in all studied thalassemic patients and controls

Parameter	Group 1a Splenctomized β-TM		Group Ib Non-splenectomized β-TM		Group II β-TI		Group III Control	
	X±SD	Range	X±SD	Range	X±SD	Range	X±SD	Range
Age (Years)	7.3±3.0	2.5–13	5.9±2.1	2.5–9	7.6±4.7	2.5–16	6.9±2.8	5–9
Sex (Male/Female)	25/15		17/9		18/8		22/18	
Weight	10.8–40.6				11–51.1			
Surface area (M^2^)	0.75±0.23*						0.98±0.25	
Hemoglobin (g/dl)	7.4±1.4**	4.8–9.6	7.3±1.3**	5.3–10	7.8±1.4**	5.9–10.3	11.5±2.2	11.1–14
Serum ferritin (μg/L)	683±160**	400–1000	483±227**	100–840	322±385**	100–1000	154±15	117–188
Total bilirubin (mg/dl)	2.3±1.1*	1.0–3.5	1.6±0.5	1.0–2.2	1.2±0.6	0.7–1.9	0.8±0.1	0.6–1.0
Total protein (g/dl)	5.6±1.3*	5.1–6.5	6.6±0.9	6.0–7.1	6.8±0.8	5.9–7.2	6.9±0.8	6.1–7.6
Albumin (g/dl)	2.3±0.6*	2.1–3.2	3.3±0.5	2.8–4.0	3.2±0.6	2.7–3.8	3.4±0.6	2.9–3.9
AST (U/L)	119±140.6**	34–466	57±40.7**	16–167	29±11	16–40	23±6.1	17–39
ALT (U/L)	189±327**	50–1352	66±35.9*	11–118	34±16.6*	11–50	19±2.6	15–24
BUN (mg/dl)	12±2.1	10–16	11±1.5	9–14	11±1.2	9–13	10±2.3	8–14
Serum creatinine (mg/dl)	0.8±0.1	0.6–1.1	0.8±0.1	0.5–0.9	0.8±0.2	0.5–1.0	0.9±0.2	0.6–1.1
Serum osmolality (mOsm/kg)	285±15.8	262–307	272±23.5	235–303	276±23.9	241–306	282±10.4	259–298
Serum HCO3 (mMol/L)	25.5±2.7*	21–29.2	25.4±2.4*	22.3–29.9	23.7±1.0*	22.6–25.2	26.9±1.7	23.5–29.4

P > 0.05: Non-significant difference

* P <0.05: Significant difference

** P < 0.01: Highly significant difference

**Table 2:** Baseline laboratory data (glomerular/tubular) of all studied thalassemic patients versus healthy controls

Parameter	Group 1a Splenctomized β-TM		Group Ib Non-splenectomized β-TM		Group II β-TI		Group III Control	
	X±SD	Range	X±SD	Range	X±SD	Range	X±SD	Range
Corrected creatinine clearance (ml/min./1.73m^2^)	70±13.5**	47–91	89±21**	57–123	97±22.6*	63–135	111±13.1	94–136
Total urinary protein (mg/g creatinine)	224±186**	18–520	180±108**	49–410	101±81.2**	97–242	43.4±9.3	24–65
Microalbuminuria (mg/g creatinine)	338±19**	8.2–94	39±23.4**	12–90	27.7±21.8**	6.0–82	11.6±5.2	4.9–17.4
NAG (Ug/g creatinine)	32±14.3**	12.3–45.4	18.3±15.2**	2.8–37.2	8.1±3.3*	4.1–14.8	6.4±1.5	3.5–9.6
RBP (mg/g creatinine)	0.67±0.7**	0.07–2.7	0.62±0.64**	0.07–1.7	0.22±0.21*	0.04–0.6	0.08±0.05	0.03–0.19
α1-microglobulin (mg/g creatinine)	27.5±13.6**	2.8–55.4	13±8.5**	4.2–37.2	7.5±2.0*	4.6–10.7	5.2±1.3	2.8–7.7
Urinary Osmolality (mOsm/kg)	522±59**	418–605	573±71.4**	470–683	601±72*	503–735	758±90	570–873
U/S osmolality	1.9±0.3**	1.4–2.9	2.1±0.3**	1.7–2.6	2.3±0.4*	1.8–2.8	2.7±0.3	2.2–3.0
% FE-HCO3	6.4±0.7**	5.0–7.6	6.1±1.7**	4.3–8.9	5.0±1.0*	3.8–6.7	3.8±0.5	3.1–5.0

P > 0.05: Non-significant difference.

* P < 0.05: Significant difference.

** P < 0.01: Highly significant difference

**Table 3:** Correlation between renal tubular markers and studied parameters in thalassemic patients

Parameter	NAG(r-values)	RBP(r-values)	α1-microglobin (r-values)
Serum ferritin (μg/L)	+0.47**	+0.43**	+0.52**
Corrected Creatinine Clearance (ml/min./1.73m^2^)	−0.40**	−0.28	−0.49**
Total urinary protein (mg/g creatinine.)	+0.60**	+0.62*	+0.64**
Microalbuminuria (mg/g creatinine)	+0.26	+0.24	+0.28

P > 0.05: Non-significant difference.

* P < 0.05: Significant difference.

** P < 0.01: Highly significant difference

**Table 4:** Frequency of renal abnormalities among thalassemic patients

Parameters (cut-off)	Number of affected patients/(total)	%
Urinary protein (> 62 mg/g creatinine)	65/92	71.0%
Microalbumin (< 22 mg/g creatinine)	25/92	29.0%
NAG (> 9.4 U/g creatinine)	53/92	58.1%
RBP (> 0.18 mg/g creatinine)	64/92	69.4%
α 1-microglobulin (> 7.8 mg/g creatinine)	50/92	54.8%
Urinary osmolality (< 578 mOsm/kg)	50/92	54.8%
U/S. osmolality (< 2.1)	59/92	64.5%
% FE- HCO3 (> 4.8%)	30/92	32.2%

**Table 5:** Comparison between different frequencies of renal abnormalities among thalassemic patients

Parameters (cut-off)		X^2^
Microalbuminuria	Total urinary protein	22.7**
NAG		2.3
RBP		0.04
α1-microglobulin		3.4
Urinary osmolality		3.4
U/S osmolality		0.4
% FE-HCO3		18.6**

P > 0.05: Non-significant difference.

* P < 0.05: Significant difference.

** P < 0.01: Highly significant difference

**Table 6:** Z-score analysis of the different studied markers for prediction of thalassemic patients with renal impairment

Parameters (cut-off)	Z-score (mean±SD)
Urinary protein (> 62 mg/g creatinine)	26.5±25.8
RBP (> 0.18 mg/g creatinine)	9.4±12.5
α 1-microglobulin (> 7.8 mg/g creatinine)	8.7±9.9
NAG (> 9.4 U/g creatinine)	8.3±9.4
Microalbuminuria (< 22 mg/g creatinine)	7.4±6.7
% FE-HCO3 (> 4.8%)	−4.1±2.5
U/S osmolality (< 2.1)	−2.6±1.2
Urinary osmolality (< 578 mOsm/kg)	2.3±1.1
